# Massive mortality of aspen following severe drought along the southern edge of the Canadian boreal forest

**DOI:** 10.1111/j.1365-2486.2010.02357.x

**Published:** 2011-06

**Authors:** Michael Michaelian, Edward H Hogg, Ronald J Hall, Eric Arsenault

**Affiliations:** Natural Resources Canada, Canadian Forest Service5320-122 Street, Edmonton, AB, Canada T6H 3S5

**Keywords:** aspen, boreal forest, climate change, dieback, drought, mortality, *Populus tremuloides*

## Abstract

Drought-induced, regional-scale dieback of forests has emerged as a global concern that is expected to escalate under model projections of climate change. Since 2000, drought of unusual severity, extent, and duration has affected large areas of western North America, leading to regional-scale dieback of forests in the southwestern US. We report on drought impacts on forests in a region farther north, encompassing the transition between boreal forest and prairie in western Canada. A central question is the significance of drought as an agent of large-scale tree mortality and its potential future impact on carbon cycling in this cold region. We used a combination of plot-based, meteorological, and remote sensing measures to map and quantify aboveground, dead biomass of trembling aspen (*Populus tremuloides* Michx.) across an 11.5 Mha survey area where drought was exceptionally severe during 2001–2002. Within this area, a satellite-based land cover map showed that aspen-dominated broadleaf forests occupied 2.3 Mha. Aerial surveys revealed extensive patches of severe mortality (>55%) resembling the impacts of fire. Dead aboveground biomass was estimated at 45 Mt, representing 20% of the total aboveground biomass, based on a spatial interpolation of plot-based measurements. Spatial variation in percentage dead biomass showed a moderately strong correlation with drought severity. In the prairie-like, southern half of the study area where the drought was most severe, 35% of aspen biomass was dead, compared with an estimated 7% dead biomass in the absence of drought. Drought led to an estimated 29 Mt increase in dead biomass across the survey area, corresponding to 14 Mt of potential future carbon emissions following decomposition. Many recent, comparable episodes of drought-induced forest dieback have been reported from around the world, which points to an emerging need for multiscale monitoring approaches to quantify drought effects on woody biomass and carbon cycling across large areas.

## Introduction

Over the past decade, major episodes of drought- and heat-related forest dieback have been reported from around the world (Allen, 2009; Allen *et al.*, 2010). This has emerged as a global concern for forests, based on model projections for continued climatic warming and widespread increases in aridity in the coming decades (Christensen *et al.*, 2007; Seager *et al.*, 2007; Rehfeldt *et al.*, 2009). Across large areas of western North America, tree mortality has already increased, likely in response to the impacts of climatic warming and drought (van Mantgem *et al.*, 2009) in combination with climatically induced, regional-scale outbreaks of bark beetles and other pests (Carroll *et al.*, 2004; Breshears *et al.*, 2005; Raffa *et al.*, 2008). Such events have far-reaching effects on forest ecosystem dynamics and local economies. Furthermore, these events can lead to large net emissions of CO_2_ to the atmosphere, as indicated by an analysis of forest carbon budgets following the unprecedented, climate-related expansion of damage by mountain pine beetle in western Canada (Kurz *et al.*, 2008). In addition to the role of drought in promoting natural disturbances such as fire and insects, it may lead more directly to subcontinental-scale reversals of forest carbon accumulation, as shown recently for the Amazon basin following an exceptionally intense drought (Phillips *et al.*, 2009).

To date, much of the global focus on drought impacts has focused on semiarid regions at low latitudes, but drought also poses a serious threat to high-latitude regions such as the boreal forests of Alaska, Canada, and Eurasia (Barber *et al.*, 2000; Hogg & Bernier, 2005; Soja *et al.*, 2007). Like the Amazon rainforest, the boreal forest has been identified as a critical ‘tipping element’ of the earth's climate system (Lenton *et al.*, 2008), where even modest increases in temperature-related water stress could lead to rapid, nonlinear changes in a wide range of biophysical processes (Hogg & Bernier, 2005). Recently, severe drought has been implicated as a major cause of transient decreases in satellite-based, model estimates of net primary productivity (NPP) for the boreal forest as a whole during 2002–2004 (Bunn *et al.*, 2007; Zhang *et al.*, 2008). However, significant challenges remain in the validation of such observations, because of (a) the relative scarcity of suitable forest monitoring networks and (b) the challenge of ‘scaling up’ ground-based measurements on heterogeneous landscapes to the generally coarser resolution of global, satellite-based observing systems.

**Fig. 1:**
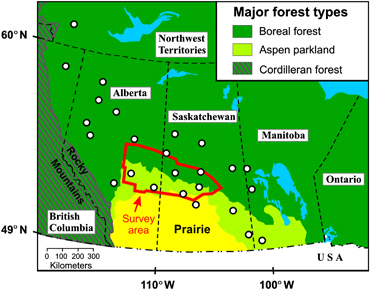
Location of drought-affected survey area (outlined in red) in western Canada. Symbols show location of the 25 CIPHA study sites (150 plots) monitored annually during 2000–2007.

This paper reports on recent massive mortality and dieback (abnormally high senescence of branches and twigs) of trembling aspen (*Populus tremuloides* Michx.) forests following a period of widespread drought in western Canada. Trembling aspen (hereafter referred to as ‘aspen’) is the most widespread tree species in North America (Perala, 1990) and is the most abundant broad-leaved tree in the Canadian boreal forest (Peterson & Peterson, 1992). Aspen is also the predominant tree in the aspen parkland (Fig. 1), a climatically drier vegetation zone where patches of fragmented forest are interspersed with prairie grassland and cropland. The climate of the aspen parkland (hereafter referred to as ‘parkland’) provides an analog for that projected for the adjacent boreal forest during this century under climate change (Hogg & Hurdle, 1995).

**Fig. 2:**
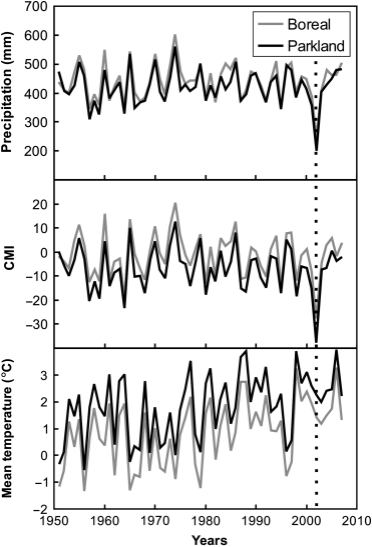
Trends in climatic variables in the survey area for successive 12-month periods ending 31 July for the years 1951–2007: (a) annual precipitation, (b) Climate Moisture Index (CMI), and (c) mean annual temperature. For each variable, average interpolated values are shown for stands located in the boreal (*N*=36) and parkland (*N*=50) portions of the survey area. Vertical dotted line shows the period of severe drought (August 2001–July 2002).

In response to public concerns about aspen dieback during the 1990s, Canadian government researchers established a study entitled ‘Climate Impacts on Productivity and Health of Aspen’ (CIPHA) that includes annual monitoring of pure aspen stands (50–90 years old) in a regional network of research plots across the west-central Canadian interior (Fig. 1). Retrospective tree-ring analysis at these plots showed that most of the temporal variation in stem growth was explained by interannual changes in moisture in combination with insect defoliation (Hogg *et al.*, 2005). Subsequent to establishment of CIPHA monitoring in 2000, the study region was affected by an exceptionally severe drought during 2001–2002 (Bonsal & Wheaton, 2005; Fig. 2). Monitoring results from the CIPHA plot network showed a regional-scale collapse in the net increment of aspen biomass following the 2001–2002 drought, because of the combined impact of a 30% decrease in stem growth and more than a twofold increase in regional stem mortality (Hogg *et al.*, 2008). Moisture was found to be the most important factor governing regional-scale, spatial variation in stand productivity, dieback, and mortality of aspen during 2000–2005.

One of the questions that has received little attention to date is how to estimate and quantify drought-induced death of tree biomass across large, heterogeneous landscapes. Such estimates are needed, for example, to assess the significance of drought as a disturbance type for national-scale reporting of forest carbon sources and sinks (e.g., Kurz *et al.*, 2009), as well as for projecting future impacts of climate change on forest ecosystem functioning and on the supply of forest products (Hogg & Bernier, 2005). A major barrier to the reliable estimation of large-scale impacts is that drought often leads to tree death patterns that are patchy across a range of spatial scales (Allen *et al.*, 2010). Such patchiness was especially evident in previous studies of aspen dieback and mortality in western Canada, where it was found that the magnitude and distribution of biomass losses across the region could not be reliably assessed solely by extrapolation from the CIPHA plot network (Hogg *et al.*, 2008).

To overcome these challenges, we conducted aerial surveys, made ground measurements at a supplementary network of temporary plots, and conducted spatial analyses of databases on land cover and meteorological records. The objectives of this paper were (a) to characterize the extent and severity of aspen dieback and mortality across a 11.5 Mha survey area (Fig. 1) where the 2001–2002 drought was most intense (Hogg *et al.*, 2008); (b) to quantify the amount of dead aspen biomass within this survey area; and (c) to examine the relationship between drought severity and its impact on the percentage and total quantity of dead biomass in aspen stands across the affected area. The results are ultimately intended to provide knowledge for projecting future impacts of climate change on the region's aspen forests and for including drought as a disturbance type in Canada's National Forest Carbon Monitoring, Accounting, and Reporting System (Kurz *et al.*, 2009).

## Materials and methods

### Study area

Spatial analyses for this study were conducted across an 11.5 Mha (115 000 km^2^) area of western Canada, bounded by latitudes 54.9–52.1°N and longitudes 113.7–104.4°W, where drought conditions were especially severe during 2001–2002 (Hogg *et al.*, 2008; Figs 1 and 2). This survey area included a 5.7 Mha (gross area) portion of the parkland and 5.8 Mha of the adjacent, climatically moister boreal forest; the boundary between these vegetation zones marks a regional, moisture-driven tipping point in ecosystem functioning (Hogg & Hurdle, 1995; Hogg & Bernier, 2005). For this study, the boundary between the boreal forest and the parkland was derived from a Canadian ecological classification (Ecological Stratification Working Group, 1996). The terrain in this area is generally flat to rolling (range of elevations 380–800 m). Mean annual temperature is 0–3 °C and mean July temperature is 15–18 °C (Hogg, 1994). Forests occupy about 25% of the total area, reflecting the intensity of agricultural land use (croplands and cattle grazing), especially in the more southerly parkland portion of the survey area. Detailed land cover was obtained from the Earth Observation for Sustainable Development of Forests (EOSD), which provides a predrought (circa year 2000) mapping of vegetation derived from Landsat TM satellite imagery (Wulder *et al.*, 2008, see Supporting Information Fxig. S1A). The fine spatial resolution (25–30 m) allowed the retention of land cover heterogeneity in the areas with patchy and fragmented forests. Our analysis was restricted to the land areas classified as broadleaf forest cover, of which about 95% is aspen. Boreal mixedwood and coniferous forests (mainly *Picea* and *Pinus*) occupied only about 5% of the total area and were not included in the current analysis (see Supporting Information Table S1 for details).

### Aerial survey of dieback and mortality

An aerial survey was conducted in August 2004 using a fixed-wing aircraft flying for 60 h at 300–400 m aboveground (see Supporting Information Fig. S1B). From the air, no distinction could be made between tree mortality and crown dieback, so these were rated together as a single measure of stand dieback. The severity of stand dieback was subjectively rated according to the following three levels: light dieback, between 20% and 35%; moderate dieback, between 36% and 55%; and severe dieback, greater than 55%. Mapping of stand dieback was aided by the use of a laptop computer containing a geographic information system (GIS) with the EOSD land cover database of broadleaf forest cover and linked to a global positioning system (GPS). Stands with overall dieback levels of <20% were considered to be healthy, i.e., below the survey threshold for mapping stand dieback. Forested areas burned by fire were also recorded during the aerial survey. In some areas, it was difficult to distinguish burned stands from drought-affected stands with severe dieback. To address this difficulty, the location of recent burns was determined from satellite-based observations of fire hotspots (details in Supporting Information: Assessment of burned areas). Discrepancies between aerial and satellite observations of areas burned were resolved by ground truthing.

### Ground-based measurements of live and dead biomass

The network of long-term, CIPHA monitoring plots has provided comprehensive, ground-based information from 25 study sites, consisting of a total of aspen 75 stands and 150 plots distributed across the parkland and southern boreal forest of west-central Canada (Fig. 1). A previous tree-ring study showed that the aspen stands in the parkland were of similar age (mean year of stand origin=1940, range from 1919 to 1959) to those in the boreal forest, whereas tree height, density and basal area were each significantly smaller in the parkland (Hogg *et al.*, 2005). Annual monitoring of the CIPHA plot network was initiated in 2000, which enabled annual reporting of mortality and aboveground biomass (live and dead) at the stand level (see Hogg *et al.*, 2008 for details on methods and study design). To estimate dead mass, we used the 2006 measurements of live and dead aspen biomass from the long-term CIPHA sites, of which six sites (including 18 stands and 36 plots) were situated within the severely drought-affected survey area. Spatial coverage across the survey area was greatly enhanced through the establishment of 397 additional temporary plots in 85 aspen-dominated stands, where the quantity of live and dead aspen biomass was determined from simple ground-based measurements during 2005–2006. Of these stands, 68 were chosen at random, under the constraint of accessibility by road, to enable the estimation of dead aspen biomass for the survey area as a whole. The remaining 17 stands were clustered nonrandomly in areas of varying dieback levels. Most of the temporary plots in the parkland were located on private land, some of which is used for cattle grazing. Stands showing significant signs of cattle grazing (primarily small patches of aspen woodland) were excluded from the sampling. At each of several random locations within each stand, a circular, variable-area plot was established. Measurements of tree diameter and height, together with biomass equations (Lambert *et al.*, 2005), were used to provide estimates of dead and live aspen biomass densities in each plot (details in Supporting Information: Estimation of dead and live biomass densities).

### Spatial interpolation of live and dead biomass

The total quantity of dead and live biomass (units in Mt) within the survey area was estimated using two different methods (details in Supporting Information: Spatial scaling of dead and live biomass). The results reported here are the more conservative estimates of dead biomass that were based on a natural neighbor interpolation (Sibson, 1981) of the 2005–2006 ground measurements within a total of 112 stands (451 plots) across the survey area. arcgis® software (Environmental Systems Research Institute, Redlands, CA, USA) was used to model the spatial variation in stand-level biomass densities (live, dead, and total) and %DEAD (dead biomass as a percentage of the total live and dead biomass) of broadleaf forests across the survey area. The EOSD broadleaf land cover map was then used as a mask to remove all other land cover types from the surface of interpolated broadleaf biomass. Live and dead biomass were then summed for broadleaf spatial units within each vegetation zone (boreal and parkland) and for the entire survey area.

### Relationship between percentage dead aspen and drought severity

Temporal and spatial variation in meteorological variables was estimated across the region using the program biosim (Régnière & Saint-Amant, 2008) and data from the national network of Environment Canada climate stations, including about 50 stations distributed within the survey area. Daily maximum and minimum temperature and total precipitation were estimated for each sampled aspen stand using an inverse distance square-weighted interpolation of data from the nearest four weather stations. The algorithm corrects for differences in latitude and elevation, as derived from a digital elevation model, between the weather stations and the sampled stands. Monthly values of mean daily maximum and minimum temperature and total precipitation were reconstructed for the sampled aspen stands over the period 1951–2007. These primary climatic variables were used to calculate a climate moisture index (CMI) that provides a measure of drought severity, here defined as the degree of water stress induced by low soil moisture. The CMI was calculated as precipitation minus potential evapotranspiration; the latter was estimated from the temperature variables (Simplified Penman–Monteith method, Hogg, 1997). The CMI was determined for successive 12-month periods ending on 31 July; this variable has been previously shown to provide a physiologically relevant indicator of drought impacts on aspen forest growth and mortality (Hogg *et al.*, 2005, 2008). Total precipitation was also used as an alternative indicator of drought severity. As indicators of heat stress at each aspen stand, we used the following thermal indicators: mean temperature, warmest mean monthly temperature, and warmest maximum daily temperature. Each of these climatic indicators was calculated for each aspen stand for successive, 12-month periods ending 31 July of the years 2000–2005. Relationships between %DEAD and the severity of annual drought or heat stress each year were examined using Pearson correlation analysis and linear regression (Zar, 1999). Measurements from the CIPHA study revealed that the standard deviation in %DEAD among adjacent stands within sites (i.e., the same climate history) was positively correlated (*r*=0.81, *P*<0.001) with mean %DEAD. Thus, the logarithmic transformation was applied to %DEAD to remove the inherent heteroscedasticity of this variable (Zar, 1999) in the analyses of relationship between %DEAD and each climatic indicator.

## Results

### Spatial distribution of dieback and dead biomass

**Fig. 3:**
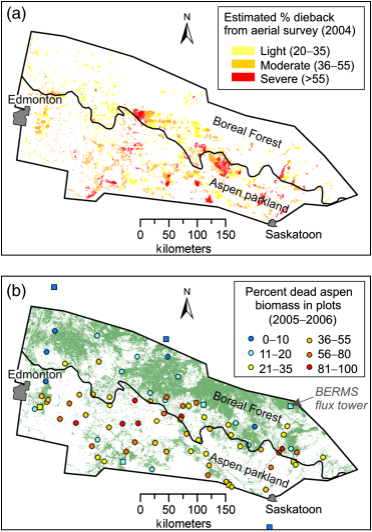
Survey area in western Canada, showing (a) gross areas of aspen forests showing dieback during aerial surveys in 2004 and (b) percent dead aspen biomass in temporary ground plots (circles) and in long-term CIPHA study areas (squares) during 2005–2006.

**Fig. 4:**
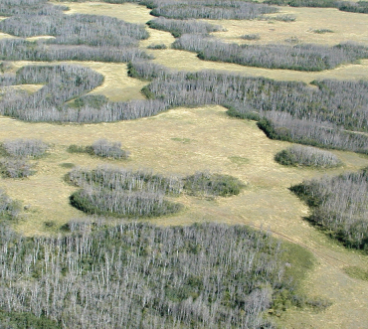
Aerial view showing an area of severe aspen mortality in the aspen parkland of Saskatchewan, Canada (photograph taken by M. Michaelian, 19 August 2004).

The 2004 aerial survey revealed extensive, patchy areas showing elevated dieback (including mortality, see Materials and methods) of aspen forests across much of the survey area (Fig. 3a and 4). The net mapped area of broadleaf forest dieback was 0.36 Mha, which excluded recent burns (affecting 0.089 Mha of broadleaf forests) and all other land cover classes (e.g., herb, wetland and coniferous forest) occurring within polygons containing fragmented patches of broadleaf forest (details in Materials and methods and Supporting Information). Dieback was most concentrated in the parkland, where 32% of the 0.62 Mha area of broadleaf forests in this zone was included within mapped areas showing significant, visible dieback. In contrast, dieback was mapped across only 10% of the broadleaf forest in the climatically moister boreal zone. Furthermore, severe dieback was mapped across 7% of the broadleaf forests in the parkland compared with only 1% in the boreal zone (Fig. 3a, further details in Supporting Information: Aerial survey). The aerially mapped areas of dieback are likely conservative, because some smaller aspen stands were missed by the aerial survey and there was a continuation of elevated mortality after 2004.

The subsequent 2005–2006 ground-based measurements in aspen stands across the survey area showed relatively high percentages of aspen stem biomass that was dead. More than 35% dead biomass was recorded in 33 of 68 randomly surveyed stands, and >80% dead biomass was recorded in six of these stands. Parallel to the aerial survey results, a predominance of this dead biomass occurred across much of the parkland and adjacent southern fringe of the boreal forest (Fig. 3b).

**Fig. 5:**
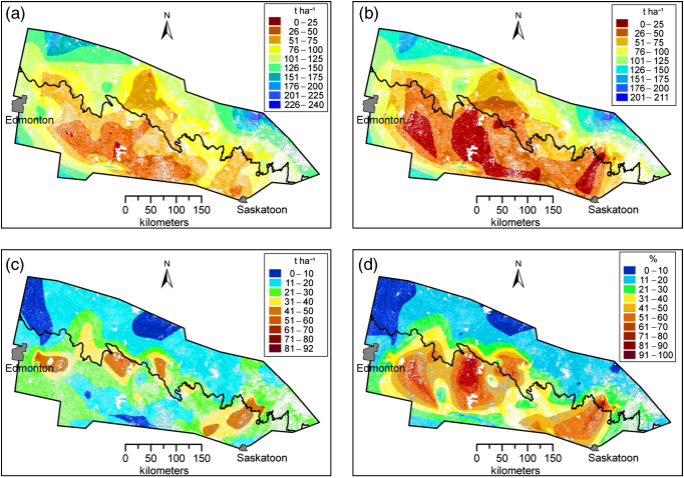
Spatial interpolations of aboveground biomass densities (tonnes per hectare) of broadleaf forest cover across the survey area, showing (a) total biomass, (b) live biomass, (c) dead biomass, and (d) %DEAD (dead biomass as a percent of total biomass). Mapped values are applicable only to the areas of broadleaf forest cover.

The mapping of spatially interpolated values from the ground plots revealed that in general, total biomass densities were greater in the boreal zone than in the parkland (Fig. 5a). This boreal–parkland difference was even more evident in the map showing live biomass densities only (Fig. 5b). Although the highest densities of dead biomass (expressed in units of tonnes per hectare) tended to be situated near the boreal–parkland boundary (Fig. 5c), areas with high %DEAD were distributed more broadly across the parkland (Fig. 5d, see also Fig. 3b).

### Estimation of dead and live biomass across the survey area

The spatial interpolation of the ground-based measurements (Fig. 5) showed that there was an estimated 45 Mt of dead aboveground biomass for broadleaf forests (predominantly aspen) in the survey area (Table 1). This quantity represented 20% of the total live and dead stem biomass of broadleaf forests in the survey area, estimated at 226 Mt. These estimates correspond to an average of 20 t ha^−1^ of dead stem biomass and 80 t ha^−1^ of live stem biomass across the total broadleaf forest area of 2.27 Mha. When calculated separately by vegetation zone (Table 1), percentage dead biomass (%DEAD, see Materials and methods) was more than twice as high in the parkland (34.6%) compared with the boreal (16.2%). However, the total amount of interpolated dead stem biomass was greater in the boreal zone (29 Mt) than in the parkland (16 Mt), owing to the much larger total stem biomass in the boreal zone (179 Mt) compared with that in the parkland (47 Mt).

**Table 1:** Total quantity of live, dead and total broadleaf forest biomass in the survey area based on the spatial interpolation of plot-based measurements

	Total (Mt)	Boreal forest (Mt)	Aspen parkland (Mt)
Live biomass	180.7	150.1	30.6
Dead biomass	45.2	29.0	16.2
Total biomass	225.9	179.1	46.8
%DEAD	20.0	16.2	34.6

Subtotals for the boreal and parkland portions of the survey area are also shown.

### Relationships between percentage dead and drought severity

Meteorological records confirmed the severity of the drought in the survey area (Fig. 2). During the 12-month period ending July 31, 2002, total precipitation was about 50% below the average annual precipitation recorded during 1951–2000. Although precipitation amounts were similar in the boreal and parkland zones, the application of a CMI indicated that conditions were considerably drier in the parkland. The CMI includes temperature effects on the rate of evapotranspiration (see Materials and methods). Both precipitation amounts and CMI values during August 2001–July 2002 indicate that this period was the driest recorded in at least 50 years. Furthermore, mean temperature for the survey showed a significant (*P*=0.00071) increasing trend of 1.8 °C during 1951–2007.

**Fig. 6:**
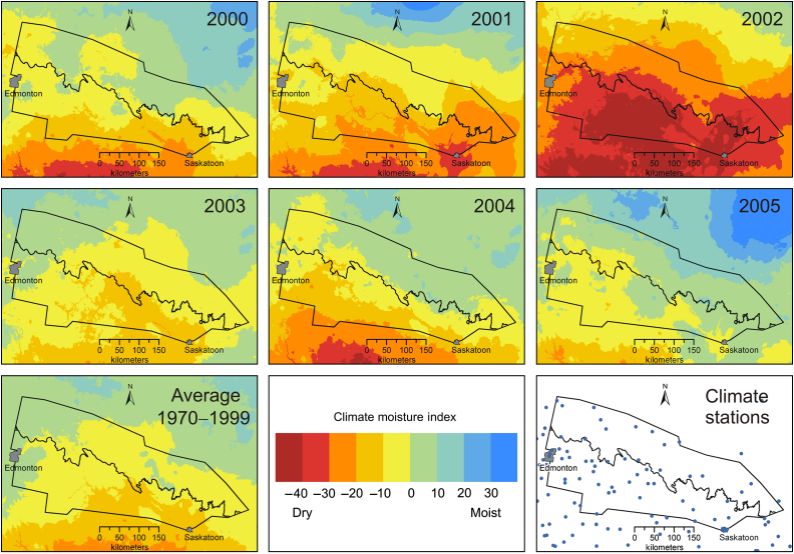
Spatial interpolation of the climate moisture index (CMI) across the survey area for 12-month periods ending 31 July of the given year (2000–2005) and for the 30-year average (1970–1999). Also shown is the distribution of climate stations used for the spatial interpolations.

The analyses of relationships between %DEAD (log_e_ transformation) and various metrics of drought and heat revealed that the strongest correlations were obtained using the minimum 12-month CMI over the period 2000–2005 (Table 2), both for aspen stands within the survey area (*r*=−0.487) and for the region as a whole (*r*=−0.625). Within the survey area, the correlation was equally strong using the 12-month CMI for the period August 1, 2001–July 31, 2002 (Table 2), when the driest conditions (lowest CMI) were recorded across the entire survey area (Figs 2 and 6). Similar results were obtained using minimum annual precipitation (2000–2005) as a drought indicator, but correlations were slightly weaker (*r*=−0.436 and −0.572 for the survey area and region, respectively). None of the correlations with indicators of heat (see Materials and methods) were as strong as those obtained for moisture. Of these, the strongest positive correlations were obtained for the warmest maximum daily temperature during 2001–2002 (*r*=0.360 and 0.513 for the survey area and region, respectively).

**Table 2:** Pearson's correlation coefficients (*r*) for relationships between log_e_-transformed values of %DEAD (measured in plots during 2005–2006) and the Climate Moisture Index (CMI, as calculated over different periods from spatial interpolation of meteorological data)

	Survey area*		Study region†	
		
Variable and period	*r*	*P*	*r*	*P*
*Annual CMI*				
2000	−0.183	0.135	−0.231	0.006
2001	−0.392	<0.001	−0.428	<0.001
2002	−0.487	<0.001	−0.622	<0.001
2003	−0.223	0.068	−0.453	<0.001
2004	−0.273	0.024	−0.299	<0.001
2005	−0.295	0.014	−0.479	<0.001
*Mean annual CMI*				
2000–2005	−0.401	<0.001	−0.545	<0.001
*Minimum annual CMI*				
2000–2005	−0.478	<0.001	−0.625	<0.001

Annual values are for 12-month periods ending on July 31 of the given year.

* Includes random temporary plots in 68 stands within the drought-affected survey area.

† Includes both random temporary plots in 68 stands and long-term CIPHA plots in 140 stands across the western Canadian interior.

**Fig. 7:**
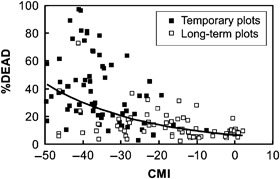
Percentage dead biomass of aspen (%DEAD) in 2005–2006 in relation to drought severity based on minimum annual value of the climate moisture index (CMI) during 2000–2005. Values are shown for stands sampled by temporary plots within the drought survey area and for stands monitored by long-term CIPHA plots across the study region. Best fitting exponential equation for all plots is also shown [%DEAD=6.6 exp(−0.0379CMI), *r*^2^=0.391, *P*<0.001].

Regression of log_e_-transformed values of %DEAD against the minimum 12-month CMI resulted in an exponential equation (Fig. 7) that gives a predicted value of 6.6% dead biomass for CMI=0 and increasing to 43.8% for CMI=−50 (corresponding to the driest conditions recorded in the survey area). As shown in Fig. 7, variation in plot-based estimates of %DEAD was highest in the most severely drought-affected areas, i.e., those with strongly negative CMI values.

### Changes in mortality and percentage dead from long-term plots

Results from the regional CIPHA network of long-term monitoring plots (Hogg *et al.*, 2008) were used to determine interannual variation in %DEAD and mortality of aspen stands within and outside the survey area (Table 3). Annual measurements showed that for the 18 CIPHA stands (six sites) within the survey area, annual mortality increased following the 2001–2002 drought and remained high (3.9–6.3%) for at least 5 years afterward. In these plots, the high mortality resulted in a major increase in %DEAD, reaching 23.5% in 2007. In contrast, the 54 CIPHA stands (18 sites) outside the survey area showed smaller increases in mortality (up to 2.2%) and %DEAD (11.0% in 2007).

**Table 3:** Changes in mean percentage dead biomass (%DEAD) and annual mortality at long-term CIPHA monitoring plots across western Canada

	Mean (SE)			
	
	%DEAD within drought survey area	%DEAD region outside drought survey area	%Mortality within drought survey area	% Mortality region outside drought survey area
*Year*				
2000	12.9 (1.5)	7.5 (0.8)	–	–
2001	13.8 (1.5)	8.2 (0.8)	1.5 (0.4)	1.1 (0.2)
2002	13.8 (1.5)	8.1 (0.8)	2.6 (0.7)	0.9 (0.2)
2003	15.9 (2.3)	8.1 (0.8)	3.9 (1.7)	1.3 (0.2)
2004	17.6 (2.9)	8.6 (0.7)	4.0 (1.5)	1.7 (0.2)
2005	19.3 (3.0)	9.6 (0.8)	5.2 (1.2)	2.2 (0.3)
2006	21.4 (3.9)	10.4 (0.9)	6.3 (2.4)	2.2 (0.3)
2007	23.5 (4.2)	11.0 (0.9)	5.3 (1.8)	1.8 (0.3)
r*	0.303	0.227	0.270	0.220
*P*	<0.001	<0.001	0.002	<0.001

Mortality is expressed as annual %loss of biomass ending in the given year. Mean values (with SE in parentheses) are shown for the 18 CIPHA stands (36 plots) located within the drought-impacted survey area (Fig. 3b) and for the 54 CIPHA stands (108 plots) in the region outside the survey area (Fig. 1), where drought impacts were less evident.

* Pearson's correlation coefficients for change in log_e_-transformed values of %DEAD or %Mortality over time (year).

Before the drought in the year 2000, the mean percentage dead biomass (%DEAD) in the CIPHA stands outside the drought survey area was 7.5 (±0.8% SE, Table 3). This value is consistent with the predicted value of 6.6% (±0.8% SE) dead biomass under relatively moist conditions (CMI=0) from the regression of 2005–2006 measurements (Fig. 7). Thus we estimated that aspen stands in the region should have about 7% dead biomass in the absence of drought or other significant disturbances.

## Discussion

Although there are many published reports of drought-induced, forest dieback from around the world (Allen *et al.*, 2010), this study is among the first to provide a quantitative estimate of climatically induced losses of forest biomass across a relatively large landscape. Our analysis covered a total survey area of 11.5 Mha area straddling the southern boundary of the Canadian boreal forest, where we recorded 45 Mt of dead broadleaf trees (primarily aspen) during 2005–2006, 4 years after an exceptionally severe drought. This dead biomass represented 20% of the total biomass (226 Mt) of broadleaf trees within the survey area. In contrast, we estimated that in the absence of severe drought, aspen stands have only about 7% dead biomass. This would correspond to an estimated baseline value of 16 Mt dead biomass, suggesting that drought led to an increase of 29 Mt in the biomass of standing dead trees across the survey area.

Significant knowledge gaps remain in current understanding of the physiological mechanisms causing tree mortality (McDowell *et al.*, 2008), especially when examining forest responses at the landscape level (Frey *et al.*, 2004). Despite the complexities of interacting factors, our results strongly implicate drought (i.e., low available soil moisture) as a major cause of the observed increase in aspen dieback and mortality. Across the survey area, we found that spatial variation of the CMI during the 2001–2002 drought was moderately well correlated with percentage dead biomass of aspen forests 4 years later (2005–2006). This is consistent with the analyses of plot-based monitoring results from the CIPHA study showing that the 2001–2002 drought was the main inciting cause of increased aspen dieback and mortality across the region (Hogg *et al.*, 2008). Comparable research by Worrall *et al.* (2010) has shown that the exceptional drought of 2001–2002 was also the major inciting factor of sudden aspen decline that has been subsequently documented across large areas of Colorado. In both regions, the observed level of drought-induced dieback and mortality was not significantly related to the age of the aspen overstory (Hogg *et al.*, 2008; Worrall *et al.*, 2010). Collectively, these results illustrate the significant threat that drought poses to North American aspen forests, even in climatically cold regions such as our survey area, where the mean annual temperature was about 1 °C before recent warming (Hogg, 1994). An emerging concern is that continued warming is expected to lead to latitudinal and altitudinal expansion of prairie-like climates, thus increasing the risk of drought-induced dieback and mortality of forests across large areas of western and central North America (Hogg & Bernier, 2005; Rehfeldt *et al.*, 2009; Frelich & Reich, 2010).

### Significance of drought to northern forest carbon cycles

The estimated quantity (29 Mt) of drought-killed biomass in the survey area corresponds to a total carbon content of about 14 Mt, based on a carbon content of 47% for aspen (Lamlom & Savidge, 2003). This is a significant carbon pool that would be equivalent to about 7% of Canada's annual carbon emissions (Environment Canada, 2007) if it were to be released in a single year; the actual timing of carbon emissions from the dead trees would depend on decomposition rates (Yatskov *et al.*, 2003). Previous experiments show that fallen aspen logs decompose more rapidly (annual mass loss of 6–8%) than other boreal tree species (Alban & Pastor, 1993; Brais *et al.*, 2006). A major knowledge gap, however, is the question of how rapidly carbon is lost from standing, drought-killed trees in relation to climatic and biotic agents, notably fungal pathogens, decay fungi, and wood-boring insects.

Overall, we consider our estimate of the drought's impact on aspen biomass and carbon cycling to be highly conservative because it excludes (a) drought-induced growth losses; (b) mortality of below ground broadleaf biomass along with mortality of other forest and vegetation types within the survey area; (c) documented drought impacts across the west-central Canadian interior outside the survey area (Hogg *et al.*, 2008); and (d) the likely role of drought in enhancing the area burned by fire (Girardin *et al.*, 2006). Furthermore, significant, ongoing elevated mortality has continued since field measurements for this analysis were made in 2005–2006 (Table S6).

### Challenges in the estimation of large-scale drought effects

Research to date has revealed inherent challenges in quantifying large-scale forest responses to extreme climatic events such as the 2001–2002 drought in western Canada. First, the impacts of drought are often amplified through interactions with other factors. For aspen, these include defoliation and stem damage by insects and fungal pathogens (Hogg *et al.*, 2002), along with a host of other variables that operate in combination with drought to trigger large-scale aspen dieback and decline (Frey *et al.*, 2004; Worrall *et al.*, 2008). In the present study, we observed a high degree of patchiness in the severity of dieback and mortality that was especially evident in the parkland zone (Figs 3 and 4). In the parkland, the fragmented character of forest cover leads to a relatively larger proportion of trees located near grassland edges, where they may be prone to increased mortality from drought, wind, and other stressors. Another important source of small-scale variation in aspen mortality arises from the clonal nature of this species, which forms patches of genetically identical trees that respond similarly to environmental stressors such as drought. Large, individual aspen clones occupying up to 1.5 ha have been documented in western Canada (Steneker, 1973), and clone sizes of >40 ha have been reported from Utah in the western US (Kemperman & Barnes, 1976).

A question not addressed by this study is how variability in falldown rates of dead trees may affect the estimation of tree mortality from surveys and measurements made at a single point in time. Results from our CIPHA monitoring plots indicate that dead aspen trees have relatively high falldown rates (about 50% after 7 years) compared with those reported for other boreal tree species (Mäkinen *et al.*, 2006; Angers *et al.*, 2010).

As this study demonstrates, spatial heterogeneity in aspen responses to drought and other stressors poses major challenges for the quantification of large-scale changes. At the stand level, intensively instrumented sites with flux towers and associated environmental measurements provide comprehensive monitoring of climate-related changes in forest ecosystem functioning, including net carbon uptake and release. For example, multiyear monitoring of a boreal aspen forest along the northern edge of our survey area (BERMS flux tower, Fig. 3b) showed that although massive mortality did not occur at this site, the drought led to 30–40% decreases in aspen leaf area and stem growth, which contributed to a collapse in net annual carbon uptake during 2004 (Krishnan *et al.*, 2006; Barr *et al.*, 2007). Such measurements, however, are expensive and currently can only provide comprehensive knowledge from relatively small areas of forest that cannot easily be extrapolated across large, heterogeneous landscapes. In contrast, global, coarse-resolution satellite observations coupled with modeling approaches have enabled monitoring of large-scale changes, including the effects of recent droughts across the circumboreal forest (Bunn *et al.*, 2007; Zhang *et al.*, 2008). Among studies of this type, however, there is currently a lack of consensus even on the fundamental question as to whether recent global change has led to ‘greening’ vs. ‘browning’ of the North American boreal forest (Alcaraz-Segura *et al.*, 2009). These issues illustrate the need for a monitoring approach that enables the integration of observations and data sets across a wide range of spatial and temporal scales.

Drought effects are most likely to occur first at semiarid ecotones (Allen & Breshears, 1998; Jump *et al.*, 2009; Mátyás, 2010) such as the transition zone from prairie to boreal forest in our study area. These regions have a high degree of land cover fragmentation, resulting in drought-induced mortality that is likely to be very patchy. This study demonstrates how drought effects can be estimated at the landscape level through the application of spatially referenced data bases (land cover and climate variables) for ‘scaling up’ observations from ground plots. These estimates could be further improved by continued development and application of integrated, remote sensing methods for finer scale mapping of forest dieback. Recent research has demonstrated the feasibility of landscape-level assessment of drought-induced increases in aspen dieback severity using multiyear time series of Landsat Thematic Mapper images (Arsenault *et al.*, 2007). We anticipate that the need for such multiscale monitoring systems will continue to increase given the recent increase in global reporting of drought-related forest dieback events with documented examples from all the world's forested continents (Allen *et al.*, 2010).

## Supporting Information

**Figure S1.** Maps of the survey area in western Canada showing (a) the satellite-based land cover classification for the survey area and (b) the location of flight lines for the aerial survey of aspen dieback.

**Table S1.** Area of EOSD land cover types found in the total survey area and in each of the boreal and parkland zones. Values in parentheses give the area of each cover type as a percentage of the respective total area or zone.

**Table S2.** Aerial survey mapped dieback areas (ha) in the total survey area and in each of the boreal and parkland zones after the application of the EOSD land cover classification mask (broadleaf classes only). Values in parentheses give the area mapped in each dieback class as a percentage of total broadleaf forest area.

**Table S3.** Ground-based mean interpolated values of percent dead biomass (%DEAD) for the aerially mapped areas in each category of dieback severity.

**Table S4.** Mean stand-level estimates (with SE in parentheses) of total, live and dead biomass density in the boreal and parkland zones within the survey area, calculated from plot-based measurements in a total of 86 aspen stands (18 CIPHA long-term stands and 68 randomly selected temporary stands). Estimates of %DEAD for each zone are calculated from mean dead biomass and mean total biomass.

**Table S5.** Total estimated quantity of live, dead and total broadleaf forest biomass in the survey area based on two methods for spatial scaling of plot-based measurements.
