# Supplementary material for: Massive mortality of aspen following severe drought along the southern edge of the Canadian boreal forest
Source: Glob Chang Biol. 2011 Jun;17(6):2084–94. doi: 10.1111/j.1365-2486.2010.02357.x (PMC3597257; doi:10.1111/j.1365-2486.2010.02357.x)
Supplement: Supplementary file 1 [file gcb0017-2084-SD1.pdf]

Supporting Information

Massive mortality of aspen following severe drought along the southern edge of the Canadian boreal forest

By Michael Michaelian, Edward H. Hogg, Ronald J. Hall and Eric Arsenault

Satellite land cover map used for the survey area

The Canadian land cover map from the Earth Observation for Sustainable Development of Forests (EOSD) project (Wulder *et al.*, 2008) was based on circa year 2000 Landsat-5 and Landsat-7 data, and was utilized in two ways for this study. First, the EOSD land cover map was used as a navigational and mapping tool for the aerial survey of aspen dieback. Second, it was used as a mask to remove nonbroadleaf forest cover types from the spatial interpolation of ground based measurements within the 11.5 Mha survey area (location shown in Fig. 1). The herb cover type, including grasslands and croplands was the dominant cover type, representing 65% of the total survey area (Table S1, Fig. S1A). Broadleaf deciduous tree cover was the dominant forest type representing 20% of the total area. Coniferous and mixedwood forest types represented only 5% of the survey area, and were mainly restricted to the boreal zone. Within the survey area, percentage total forest cover was greater in the boreal zone (38%) than in the parkland zone (12%). Not only was there less forest cover in the parkland but its spatial distribution was also much more fragmented.

Table S1 Area of EOSD land cover types found in the total survey area and in each of the boreal and parkland zones. Values in parentheses give the area of each cover type as a percentage of the respective total area or zone.

Land cover type	Total survey area		Boreal zone		Parkland zone	
	ha	(%)	ha	(%)	ha	(%)
Herb	7,482,089	(65)	2,776,199	(48)	4,705,890	(82)
Broadleaf forest	2,276,005	(20)	1,652,029	(29)	623,976	(11)
Coniferous forest	364,623	(3)	321,683	(6)	42,940	(1)
Mixedwood forest	240,441	(2)	234,821	(4)	5,620	(<1)
Shrub	118,381	(1)	110,468	(2)	7,913	(<1)
Wetland	347,645	(3)	289,514	(5)	58,131	(1)
Water	546,319	(5)	346,309	(6)	200,010	(4)
Other*	116,078	(1)	48,838	(1)	67,240	(1)
Total area	11,491,580	(100)	5,779,860	(100)	5,711,720	(100)

*Includes exposed, cloud and shadow

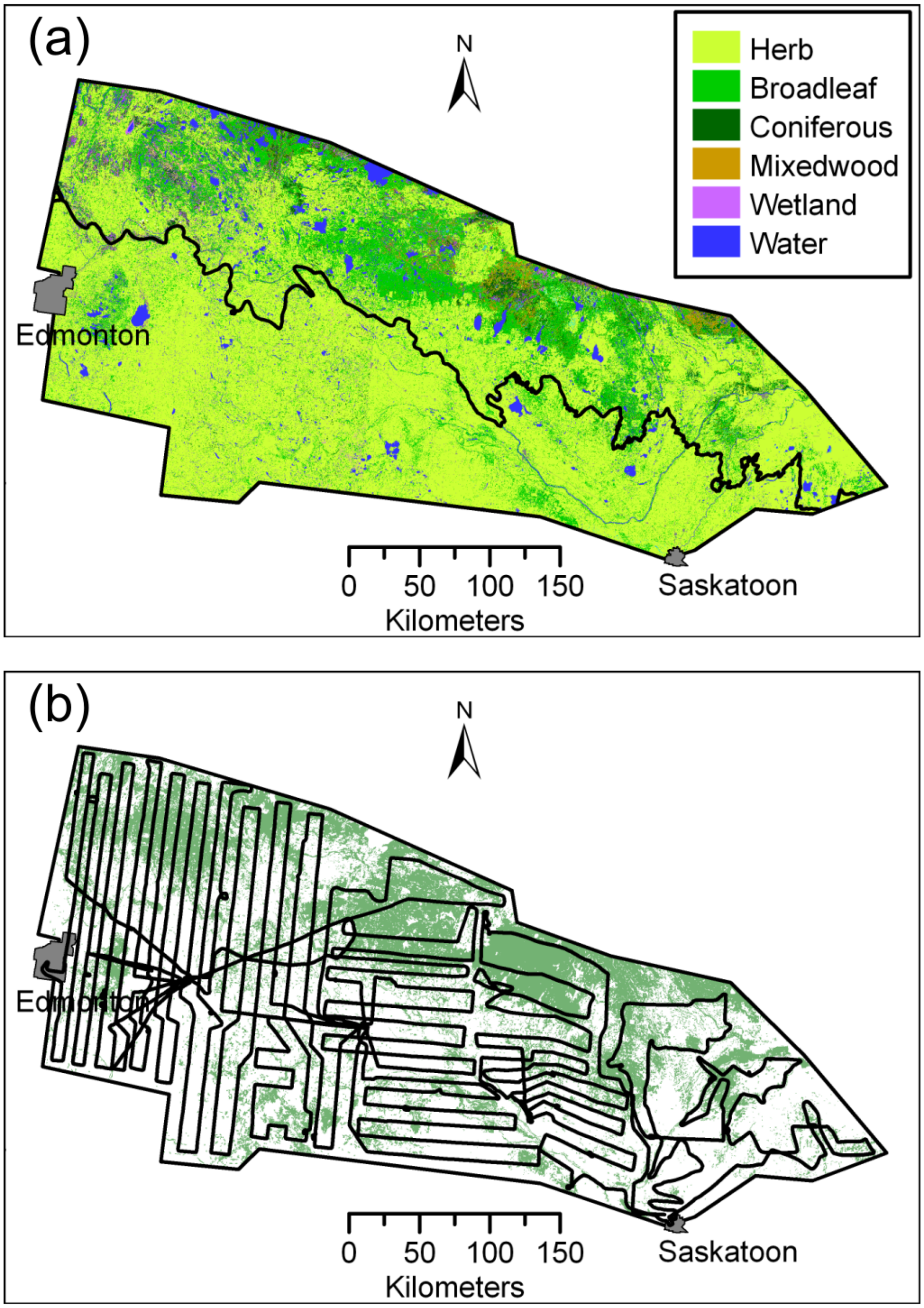

Fig. S1 Maps of the survey area in western Canada showing (a) the satellite-based land cover classification for the survey area and (b) the location of flight lines for the aerial survey of aspen dieback.

Ground validation of the EOSD land cover product

A total of 519 ground plots, located within or near the survey area, were used to validate the EOSD land cover product. These included the plots used in the spatial interpolation and an additional 68 plots where related research was conducted. The species composition of each plot was compared to the land cover type assigned to the associated EOSD pixels. A 40-m buffer was constructed around each plot center to account for positional errors of both the EOSD pixels and the global positioning system (GPS) that was enabled by a Wide Area Augmentation System (WAAS). Cover match was assessed as presence or absence of the appropriate cover type within the plot buffer.

The field survey results indicated that the appropriate land cover type (broadleaf forest) was present within the 40-m buffer for 87% of the plots. Another 4% of the plots were classified as mixedwood (i.e., forests with a mixture of broadleaf and coniferous trees), and only 9% were incorrectly classified as having a non-forest land cover type. As observed from the aerial survey, the EOSD land cover appeared to be accurate in representing the quantity and distribution of broadleaf forest cover. The EOSD land cover map was therefore a good representation of the actual broadleaf ground cover as assessed from both ground-based and aerial observations.

Aerial survey

The aerial survey of broadleaf forest dieback was conducted during 8–28 August 2004, and covered a linear distance of approximately 11,000 km with flight lines about 10 km apart across most of the 11.5 Mha survey area (Fig. S1B). A mobile unit was used that combined the capabilities of global positioning (GPS) with the MapInfo® (Pitney Bowes Business Insight, North Greenbush, NY. USA) geographic information systems (GIS). The unit was configured with the EOSD broadleaf land cover map to assist with the in-flight navigation and mapping of polygons on the landscape containing broadleaf forest. This enabled accurate positioning of dieback areas and proved to be indispensable considering the high degree of forest fragmentation in the survey area. During the flight, broadleaf forests with visible dieback (including mortality) were mapped in three categories of visible dieback severity: light, 20–35%; moderate, 36–55%; and severe, >55%. These polygons typically contained patches of broadleaf forest interspersed with other cover types (Fig. 4, Fig. S1A); consequently, the application of the EOSD broadleaf mask resulted in reductions of 70–80% in the estimated areas of dieback relative to the area of mapped polygons. The percentage of broadleaf forest area showing dieback in each category was consistently greater in the parkland than in the boreal zone (Table S2).

Table S2 Aerial survey mapped dieback areas (ha) in the total survey area and in each of the boreal and parkland zones after the application of the EOSD land cover classification mask (broadleaf classes only). Values in parentheses give the area mapped in each dieback class as a percentage of total broadleaf forest area.

Dieback severity (%)	Total survey area		Boreal zone		Parkland zone	
	ha	(%)	ha	(%)	ha	(%)
Nil (0 to 19)	1,914,647	(84)	1,490,781	(90)	423,866	(68)
Light (20 to 35)	151,941	(7)	89,088	(5)	62,853	(10)
Moderate (36 to 55)	143,131	(6)	50,860	(3)	92,271	(15)
Severe (>55)	66,286	(3)	21,300	(1)	44,986	(7)
Total broadleaf forest	2,276,005	(100)	1,652,029	(100)	623,976	(100)

Ground validation of aerial survey

The 2005−2006 ground plot measurements were used to validate the 2004 aerial survey. Stand locations were determined by averaging each GPS-derived plot coordinate within the stands. The ground derived measures of crown dieback and mortality were combined to a single measure of overall dieback, expressed as stand-level percentage dead biomass (%DEAD, see section on ground plot measurements, below). The following two methods were used to validate the results of the aerial survey against the ground-based measure of %DEAD:

1. For the first validation, each stand coordinate was buffered by 250 m, which was the estimated positional error of the aerial survey polygon placement. These buffers were overlaid with the aerial survey dieback polygons and their aerial ratings of dieback severity were extracted. Fifty-nine of these buffered stands intersected with the aerial survey dieback polygons. Where more than one aerial survey polygon was intersected by the buffer, the extracted severity was that of the largest polygon. A Pearson correlation analysis showed that there was a moderate, significant correlation ($r = 0.58$, $p < 0.0001$) between the ground measured %DEAD and the aerial dieback ratings.

2. For the second validation, 10,000 sampling points were randomly selected from the areas in the EOSD broadleaf land cover type. From each random point, values were extracted from the interpolated surface of %DEAD surface and the aerial classification of dieback. Mean values of %DEAD were then determined for each category of aerially mapped dieback (nil, light, moderate, and severe). The results showed the expected, progressive increase in mean %DEAD across the aerially mapped categories of dieback from nil to severe (Table S3).

Table S3 Ground-based mean interpolated values of percent dead biomass (%DEAD) for the aerially mapped areas in each category of dieback severity.

Dieback severity (%)	Mean %DEAD	SD
Nil (<20)	23.1	13.2
Light (20 to 35)	31.8	12.6
Moderate (36 to 55)	35.5	14.7
Severe (>55)	41.6	17.6

The comparison between these ground and aerially based measures of dieback reflects the differing perspectives of each approach. First, stands with high levels of branch dieback but low tree mortality would be rated as severe in the aerial survey but because the stems are alive, they would have a low or moderate %DEAD as measured from the ground. Second, the interpolation of ground-based measurement tends to smooth the spatial variability, removing the extreme high and low values of %DEAD. Third, there was a 1- to 2-year time lag between the aerial survey and the ground plot measurements during a period when mortality rates remained relatively high (see below). Finally, the conservative aerial survey detection threshold of 20% and the fact that dead stems and branches of subdominant trees are not easily visible from the air may result in undetected dieback and mortality. For example, mean %DEAD was estimated at 23.1% across the large proportion (84%, Table S2) of broadleaf forest area in the "nil" category, which had a subjective aerial rating of <20% dieback. These factors highlight the informational value of integrated systems that incorporate both field and aerial or remotely sensed monitoring compared to those that may use only one of these approaches.

Assessment of burned areas

From the air, it was not always possible to discriminate drought-killed aspen stands from those burned by fire. To assist in the identification of burned areas, we obtained remotely sensed historic fire data. Moderate Resolution Imaging Spectroradiometer (MODIS) hotspot data were obtained for the period November 2000 to November 2004 and compiled into a single composite. These data were collected by NASA's Terra and Aqua satellites using mid and thermal infrared (IR) sensors (NASA/University of Maryland, 2005). The hotspot data provided locations (but not area burned) of past fires at a relatively coarse (1 km pixel) resolution. In cases where there were discrepancies in the interpretation of burned areas as mapped from the aerial survey and the MODIS hotspot composite, ground surveys were conducted to search for evidence (e.g., charring of woody material) of recent fire.

There was generally good agreement between fire areas mapped from the air and the MODIS hotspot composite: areas mapped as burned had an average of 0.46 hotspot points per km^2 compared to an average of 0.01 per km^2 for areas mapped as having dieback but not fire. Discrepancies occurred where fire may have occurred before November 2000, or where the burning of slash piles during land clearing of adjacent stands resulted in MODIS hotspots that resembled forest fires. Most of the hotspots

occurring in areas mapped as unburned and having dieback were of this nature. Several aspen stands showed signs of both dieback and fire when checked during the ground survey. A gross burned area of 181,226 ha was mapped during the aerial survey, and the majority of the burns occurred in the boreal forest rather than the parkland. This area increased slightly to 203,697 ha after using our ground verifications to correct the discrepancies between the aerial survey and the MODIS hotspot composite. When the EOSD broadleaf mask was applied (to exclude other land cover types), the net area of burned broadleaf forest was 89,524 ha, corresponding to 4% of the total broadleaf area.

Estimation of live and dead biomass densities from ground plot measurements

Estimates of live and dead biomass densities (units of tonnes per hectare) were made at 451 temporary and long-term plots in 112 aspen stands across the survey area. These included 54 long-term monitoring plots (27 stands) from the CIPHA study (Hogg *et al.*, 2008), situated within or near the survey area.

Each CIPHA plot was rectangular, with a width of 10 m and variable length (typically 15–25 m) to include at least 25 living aspen trees when plots were established in 2000. The status (live or dead) and the percent crown dieback of each tree in the CIPHA plots were determined as part of the annual health assessment of 2006. Biomass of each live and dead tree was estimated using height and diameter (at 1.3-m stem height) measurements taken in 2004 (method described below). Biomass densities of live and dead trees within each plot was calculated by dividing total tree biomass by plot area, stand-level biomass density was estimated as the average of the two plots sampled within each stand (Hogg *et al.*, 2008).

At each of the 397 temporary plots established in 2005 and 2006, live and dead stem biomass was determined within a circular, variable area plot using a wedge prism (Avery & Burkhart, 2002) with a basal area factor of 2 m^2 ha^{-1}. This sampling approach resulted in the inclusion of approximately 10–20 trees per plot and enabled stand-level estimates of live and dead basal area (in units of square meters per hectare). The species, status (live or dead) and percent crown dieback were recorded for each tree in all plots. Individual tree heights and diameters (at 1.3-m height) were measured in approximately half of the plots and average (modal) diameters were measured in remaining plots (2005 temporary plots).

For both temporary and long-term plots, above-ground stem biomass was calculated using a set of Canadian biomass equations for aspen (Lambert *et al.*, 2005). There were two equation forms used; one that used both height and diameter measurements and another that used only diameter. Where height was available the equation chosen included both height and diameter parameters. Otherwise (e.g., dead trees with snapped tops and the 2005 temporary plots), we used the diameter-based equations. The effect of using modal stem diameters instead of individual diameters (2005 temporary plots) was small because biomass depended largely on basal area, which was measured directly by the prism sweeps. Furthermore, most aspen stands had relatively homogeneous ages and stem diameters.

For each tree in temporary and long-term plots, biomass was partitioned into stem, bark and branch components (leaf biomass was excluded). Stem and bark biomass was

classified as living or dead according to the tree's status. Branch biomass was partitioned as live or dead using the field-rated percentage of crown dieback.

For stands sampled by the temporary, variable area plots, aboveground biomass density (*M*, expressed in units of tonnes per hectare) was calculated as:

$$M = \sum_{i}^{n} m_i / a_i \times A / n$$

where: m_i = live or dead biomass of tree$_i$ (stem + bark + branch biomass, tonnes)

a_i = basal area of tree$_i$ (square meters)

n = total number of trees sampled from the wedge prism sweeps

A = stand basal area (square meters per hectare) as measured by the wedge prism sweeps.

The variable %DEAD (dead biomass as a percentage of total living plus dead biomass) was then calculated for all stands sampled by the temporary and long-term plots.

Results of these ground-based measurements (Table S4) showed that in general, the stands in the parkland had greater mean %DEAD than those in the boreal forest. This difference reflected the greater dead biomass density and smaller live biomass density of aspen-dominated forests in the climatically drier parkland zone. At the stand level, %DEAD encompassed nearly the full range of possible values in both zones (3–96% for boreal stands and 6–97% for parkland stands).

Table S4 Mean stand-level estimates (with SE in parentheses) of total, live and dead biomass density in the boreal and parkland zones within the survey area, calculated from plot-based measurements in a total of 86 aspen stands (18 CIPHA long-term stands and 68 randomly selected temporary stands). Estimates of %DEAD for each zone are calculated from mean dead biomass and mean total biomass.

		Biomass			
Zone	Number of stands	Total ($t\ ha^{-1}$)	Live ($t\ ha^{-1}$)	Dead ($t\ ha^{-1}$)	Estimated %DEAD
Boreal	50	106.1 (7.2)	82.2 (7.4)	23.9 (2.2)	22.6
Parkland	36	81.2 (5.8)	51.4 (5.5)	29.8 (2.9)	36.6

Spatial scaling of dead and live biomass

Two methods were used to estimate the total quantity of dead, live and total aboveground biomass (units in Mt) of broadleaf forests within the survey area (Table S5). The first method was to apply a simple scaling procedure in which the mean stand-level values of these variables in each zone (boreal or parkland, Table S4) were multiplied by the total broadleaf forest area in the corresponding zone (Table S1). The second method, described in Methods (main text of this paper), was based on a spatial interpolation of the measured, stand-level values of dead and total (dead + live) biomass density across the survey area (Fig. 5).

The results showed that the total estimated quantity of dead aboveground biomass in the survey area was 58 Mt using the simple scaling method and 45 Mt using the spatial interpolation method (Table S5). The latter, more conservative estimate is reported in the main text of this paper. We considered this estimate to be more appropriate because a) it retains local spatial heterogeneity of the plot-based measurements, b) it better reflects climatically induced gradients in stand characteristics within each zone and c) it minimizes the underweighting of the less numerous, healthier plots in the northern portion of the boreal zone, where road access was limited. The result of the interaction of these factors is that the simple scaling procedure overestimated the total dead biomass of the boreal by 37% relative to that derived from the spatial interpolation (Table S5).

Table S5 Total estimated quantity of live, dead and total broadleaf forest biomass in the survey area based on two methods for spatial scaling of plot-based measurements.

	Biomass in survey area			
Method	Total (Mt)	Live (Mt)	Dead (Mt)	Estimated %DEAD
Simple scaling	226.0	167.9	58.1	25.7
Spatial interpolation	225.9	180.7	45.2	20.0

In contrast to the results for dead broadleaf biomass, the two methods gave nearly identical estimates of 226 Mt for total broadleaf biomass in the survey area (Table S5). We assessed the reliability of these estimates through a comparison with an independent data set of broadleaf forest wood volume from the EOSD/NFI National Biomass Mapping program that incorporates specific parameters relating to ecological zonation, stand age, forest type, density and crown closure (Hall *et al.*, 2008). The broadleaf volume estimate was converted into biomass using the reported mean wood density of trembling aspen (0.37 t m^{-3} for dry biomass to green volume, Peterson & Peterson, 1992). The resulting estimate of 235 Mt for total broadleaf biomass, as calculated above from the EOSD mapping project (Hall *et al.*, 2008), was within 5% of our estimates based on the plot-based measurements (Table S5).

References

Avery TE, Burkhart HE (2002) *Inventories with point samples in forest measurements*, 5th ed., pp. 230–258, McGraw-Hill, New York, NY, USA.

Hall RJ, et al. (2008) *Taking stock of Canada's forests for Canadians: mapping biomass carbon stocks with satellite remote sensing data*. Project final report to Canadian Space Agency. Natural Resources Canada, Canadian Forest Service.

Hogg EH, Brandt JP, Michaelian M (2008) Impacts of a regional drought on the productivity, dieback and biomass of western Canadian aspen forests. *Canadian Journal of Forest Research*, **38**, 1373–1384.

Lambert M-C, Ung C-H, Raulier F (2005) Canadian national tree aboveground biomass equations. *Canadian Journal of Forest Research*, **35**, 1996–2018.

NASA/University of Maryland (2005) *MODIS Active Fire Detections*. Data set. MODIS Rapid Response Project, NASA/GSFC [producer], University of Maryland, Fire Information for Resource Management System [distributors]. Available at http://maps.geog.umd.edu [accessed 27 April 2010].

Peterson EB, Peterson NM (1992) *Ecology, management, and use of aspen and balsam poplar in the Prairie Provinces*, Canada. Special Report 1. Forestry Canada, Northwest Region, Northern Forestry Centre, Edmonton, AB, Canada.

Wulder MA, White JC, Cranny MM *et al.* (2008) Monitoring Canada's forests. Part 1: Completion of the EOSD land cover project. *Canadian Journal of Remote Sensing,* **34**, 549–562.
